# Three Novel Biphenanthrene Derivatives and a New Phenylpropanoid Ester from *Aerides multiflora* and Their *α*-Glucosidase Inhibitory Activity

**DOI:** 10.3390/plants10020385

**Published:** 2021-02-17

**Authors:** May Thazin Thant, Boonchoo Sritularak, Nutputsorn Chatsumpun, Wanwimon Mekboonsonglarp, Yanyong Punpreuk, Kittisak Likhitwitayawuid

**Affiliations:** 1 Department of Pharmacognosy and Pharmaceutical Botany, Faculty of Pharmaceutical Sciences, Chulalongkorn University, Bangkok 10330, Thailand; maythazinthant@mohs.edu.mm (M.T.T.); Kittisak.L@chula.ac.th (K.L.); 2 Department of Pharmacognosy, University of Pharmacy, Yangon 11031, Myanmar; 3 Natural Products for Ageing and Chronic Diseases Research Unit, Faculty of Pharmaceutical Sciences, Chulalongkorn University, Bangkok 10330, Thailand; 4 Department of Pharmacognosy, Faculty of Pharmacy, Mahidol University, Bangkok 10400, Thailand; nutputsorn.cha@mahidol.ac.th; 5 Scientific and Technological Research Equipment Centre, Chulalongkorn University, Bangkok 10330, Thailand; wanwimon.m@chula.ac.th; 6 Department of Agriculture, Kasetsart University, Bangkok 10900, Thailand; cyyp01@hotmail.co.th

**Keywords:** *Aerides multiflora*, Orchidaceae, *α*-glucosidase inhibition, biphenanthrene derivatives, phenylpropanoid ester

## Abstract

A phytochemical investigation on the whole plants of *Aerides multiflora* revealed the presence of three new biphenanthrene derivatives named aerimultins A–C (**1**–**3**) and a new natural phenylpropanoid ester dihydrosinapyl dihydroferulate (**4**), together with six known compounds (**5**–**10**). The structures of the new compounds were elucidated by analysis of their spectroscopic data. All of the isolates were evaluated for their *α*-glucosidase inhibitory activity. Aerimultin C (**3**) showed the most potent activity. The other compounds, except for compound **4**, also exhibited stronger activity than the positive control acarbose. Compound **3** showed non-competitive inhibition of the enzyme as determined from a Lineweaver–Burk plot. This study is the first phytochemical and biological investigation of *A. multiflora*.

## 1. Introduction

Diabetes mellitus (DM) is one of the main causes of global morbidity and mortality [1]. The disease is caused by insufficient insulin secretion and/or action. DM is associated with high blood glucose levels, and type 2 DM is the most common form, covering 90–95% of all diabetes cases [2]. Drugs currently used for treating DM can be classified into several classes following their chemical structures and modes of action, and some have limitations due to their adverse reactions or unpleasant effects [3]. *α*-Glucosidase is an enzyme located in the small intestine. It is responsible for converting starch and disaccharides into monosaccharides (glucose). Inhibition of this enzyme can significantly reduce postprandial hyperglycemia [4]. *α*-Glucosidase inhibitors (AGIs) have been widely used in combination with other anti-DM drugs in the management of type 2 DM [5,6,7,8].

However, AGIs can cause liver injuries and gastrointestinal side effects [9,10]. There has been a growing interest in developing antidiabetic drugs of botanical origin because they are perceived as possessing fewer undesired effects [11,12]. Several promising AGIs have been reported from some members of the family Orchidaceae, such as *Dendrobium tortile* [13], *Bulbophyllum retusiusculum* [14], and *Arundina graminifolia* [15].

*Aerides* is a small genus of epiphytes in the family Orchidaceae. It consists of approximately 21 species that are native to South and South-East Asia [16]. Some *Aerides* species have been used in traditional medicine. For example, *Aerides falcata* has been used for boosting the immune system, whereas *Aerides odoratum* has been known for its antibacterial properties [17]. Phytochemical screening of *Aerides odoratum* suggested the presence of alkaloids, glycosides, flavonoids, saponins, tannins, terpenoids, steroids, and anthroquinones [18]. Several phenanthrene derivatives have been identified from *Aerides rosea* [19] and *Aerides crispum* [20].

**Figure 1:**
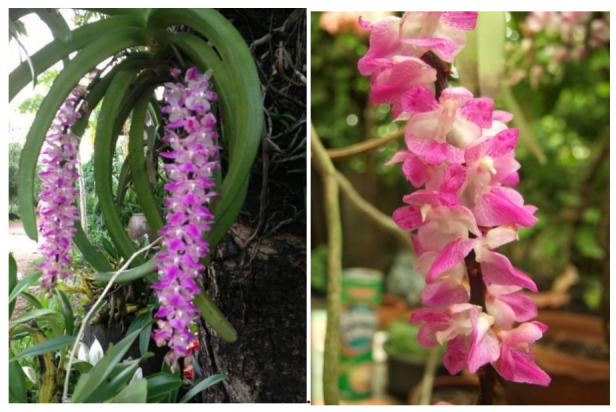
*Aerides multiflora* Roxb.

*Aerides multiflora* Roxb. (Figure 1) is commonly known as “The Multi-flowered Aerides” [21] and called “Malai Dang” in Thai [22]. It has several synonyms, including *Aerides affinis*, *Aerides godefroyana*, *Aerides lobbii*, *Cleisostoma vacherotiana*, and *Epidendrum geniculatum* [23]. The plant is indigenous to Bangladesh, India, Nepal, Myanmar, Thailand, Malaysia, Philippines, Laos, Cambodia, and Vietnam. *A. multiflora* has been traditionally used as a tonic [24]. It has also been used to treat cuts and wounds [17,25] and fractured and dislocated bones [26]. In an earlier study, its tubers showed an antibacterial effect in vitro [27]. As a continuation of our investigation of orchids for *α*-glucosidase inhibitors [28,29,30], a MeOH extract obtained from the whole plants of *Aerides multiflora* was evaluated and found to possess strong inhibitory property against the enzyme (82.4 ± 9.5% inhibition at 100 µg/mL). In this communication, we describe our findings on the chemical constituents of this plant and their *α*-glucosidase inhibitory activity.

## 2. Results and Discussion

### 2.1. Structural Characterization

**Figure 2:**
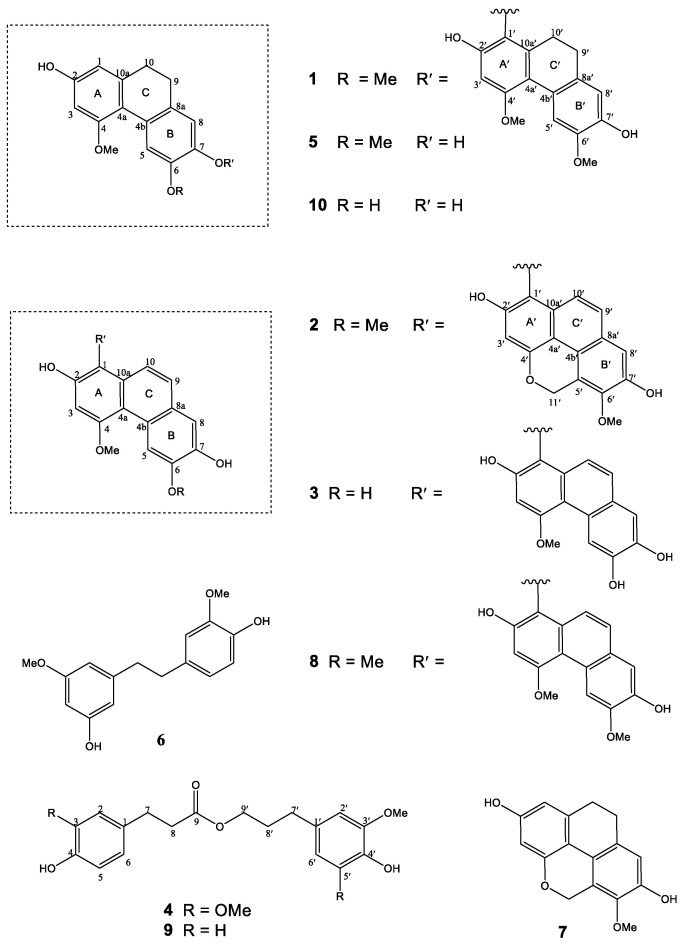
Chemical structures of compounds **1**–**10** isolated from *Aerides multiflora.*

A total of 10 polyphenolic compounds were isolated from the MeOH extract of *Aerides multiflora* through solvent partition and repeated chromatography. They were characterized as three unknown compounds, named aerimultins A-C (**1**–**3**) and a new natural product, dihydrosinapyl dihydroferulate (**4**), together with six known compounds, i.e., 6-methoxycoelonin (**5**) [31], gigantol (**6**) [32], imbricatin (**7**) [33], agrostonin (**8**) [34], dihydroconiferyl dihydro-*p*-coumarate (**9**) [35] and 5-methoxy-9,10-dihydro- phenanthrene-2,3,7-triol (**10**) [36] (Figure 2).

Compound **1** was isolated as a whitish-brown amorphous solid. It showed a [M+Na]^+^ at *m*/*z* 565.1841 (calculated for C_32_H_30_O_8_Na, 565.1838) in the HR-ESI-MS. The IR spectrum showed absorption bands for hydroxyl (3350 cm^−1^), aromatic ring (2923, 1605 cm^−1^), methylene (1462 cm^−1^) and ether (1221 cm^−1^) groups. The UV absorptions at 265, 305, and 315 nm were indicative of a dihydrophenanthrene skeleton [37]. The ^13^C NMR and HSQC spectra revealed signals for twenty-four aromatic carbons, plus eight aliphatic carbons representing four methoxy and four methylene groups. The four CH_2_ carbons at 29.0 (C-9), 31.4 (C-10), 29.9 (C-9′), and 24.1 (C-10′) displayed HSQC correlations to the protons at δ 2.45 (2H, m, H_2_-9) and 2.56 (2H, m, H_2_-10) and 2.52 (4H, br s, H_2_-9′, H_2_-10′), respectively. These NMR signals suggested that **1** should be a dimeric compound consisting of two units of 9,10-dihydrophenanthrene (Table 1). The first unit of **1** (rings A, B, and C) should be derived from methoxycoelonin (**5**), a dihydrophenanthrene also obtained in this study, because its ^1^H and ^13^C NMR properties bore a close resemblance to those of **5** (Table 1). For example, in ring A of the first unit of **1**, the proton at C-1 (δ 6.35, 1H, d, *J* = 2.5 Hz) exhibited HMBC correlation with C-10 (δ 31.4) and NOESY interaction with H_2_-10. H-3 (δ 6.46, 1H, d, *J* = 2.5 Hz) of **1** showed a NOESY cross peak with MeO-4 protons (δ 3.89, 3H, s). The hydroxyl proton at C-2 was observed at δ 8.35 (1H, s). For ring B of **1**, the following ^1^H NMR signals were found: two one-proton singlets at δ 6.33 (1H, s, H-8) and 7.98 (1H, s, H-5), and a three-proton singlet at δ 3.92 (3H, s, MeO-6) which showed a NOESY cross-peak with H-5. The second unit of **1** (rings A′, B′, and C′) also exhibited ^1^H and ^13^C NMR data similar to those of **5** (Table 1). For instance, the ^1^H NMR spectrum of **1** exhibited two singlet proton signals at 6.66 (1H, s, H-8′) and 7.93 (1H, s, H-5′), two methoxy groups at δ 3.84 (3H, s, MeO-6′) and 3.91 (3H, s, MeO-4′), and two hydroxyl groups at δ 7.44 (s, HO-7′) and 8.25 (s, HO-2′). The HMBC spectrum of **1** showed correlation from H-3′ to C-1′ (δ 133.7) and C-4a′ (δ 117.1), and from H-5′ to C-4a′ (δ 117.1), C-8a′ (δ 131.4) and C-7′ (δ 145.6). H-8′ (δ 6.66, 1H, s) displayed HMBC correlation with C-9′ (δ 29.9) and NOESY interaction with H_2_-9′. The methoxyl protons at C-4′ (δ 155.3) and C-6′ (δ 146.1) showed NOESY correlations with H-3′ and H-5′, respectively. However, the second dihydrophenanthrene unit of **1** showed the absence of a H-1′ signal, with the signal for H-3′ appearing as a singlet at δ 6.65. Moreover, in the ^13^C NMR spectrum of **1**, the signal for C-1′ of this unit was downfield shifted and observed as a quaternary carbon at δ 133.7, with HMBC correlations with H-3′ (δ 6.65, s), H_2_-10′ (δ 2.52, br s) and HO-2′ (δ 8.25, s). These NMR properties indicated that the structure of **1** consisted of two methoxycoelonin (**5**) units connected to each other through an ether linkage at C-7 and C-1′. This was also supported by the absence of a hydroxyl proton at C-7 (δ 146.6). Based on the above spectral data, compound **1** was characterized as a new dimeric 9,10-dihydrophenanthrene derivative and given the trivial name aerimultin A.

Compound **2**, a brown amorphous solid, exhibited [M+Na]^+^ at *m*/*z* 559.1376 (calculated for C_32_H_24_O_8_Na, 559.1368) in the HR-ESI-MS, corresponding to the molecular formula C_32_H_24_O_8_. The IR spectrum showed absorption bands due to the presence of hydroxyl (3368 cm^−1^), aromatic ring (2919, 1587 cm^−1^) and ether (1259 cm^−1^) functionalities. The UV absorptions at 265, 315.5 and 370 nm were suggestive of a phenanthrene skeleton [30]. Compound **2** should be a dimeric phenanthrene, as suggested from the ^1^H NMR signals for two pairs of *ortho*-coupled doublets, representing H-9 (δ 7.36, 1H, d, *J* = 9.5 Hz), H-10 (δ 6.98, 1H, d, *J* = 9.5 Hz), H-9′ (δ 7.37, 1H, d, *J* = 9.0 Hz), and H-10′ (δ 6.92, 1H, d, *J* = 9.0 Hz) (Table 2). The first phenanthrene unit of **2** (rings A, B, and C) exhibited ^1^H and ^13^C NMR resonances similar to those of agrostonin (**8**), a biphenantherene also isolated from this plant (Table 2). These included three one-proton singlets at δ 6.99 (1H, s, H-3), 7.19 (1H, s, H-8) and 9.24 (1H, s, H-5), and two methoxy groups at δ 4.06 (3H, s, MeO-6) and 4.22 (3H, s, MeO-4). The proton at C-8 showed HMBC correlation with C-9 (δ 126.5). The protons H-3 and H-5 exhibited three-bond couplings to C-4a (δ 116.2) in the HMBC spectrum. The NOESY correlations of the MeO-4 and MeO-6 protons with H-3 and H-5, respectively, supported the attachment of these methoxy groups at C-4 and C-6. The quaternary carbon at δ 109.3 was assigned as C-1 according to its HMBC cross-peaks with H-3 and H-10. For the second phenanthrene unit (rings A′, B′, and C′), the presence of oxymethylene protons at δ 5.79 (2H, d, *J* = 1.5 Hz, H_2_-11′) indicated a phenanthropyran structure [38]. The ^1^H NMR spectrum also displayed two sharp one-proton singlets at δ 6.81 (1H, s, H-3′) and 7.21 (1H, s, H-8′), and a methoxy group at δ 3.95 (3H, s, MeO-6′). The assignments of H-8′ and H-3′ were supported by their HMBC correlations with C-9′ (δ 127.9) and C-1′ (δ 110.2), respectively. The HMBC correlations of C-6′ (δ 144.2) with MeO-6′ protons and H_2_-11′ indicated the location of the methoxy group at C-6′. The C-1′ of this second unit showed HMBC correlations with H-3′ and H-10′. The chemical shifts of C-1 (δ 109.3) and C-1′ (δ 110.2) suggested that they were not oxygenated, but, instead, they formed a C−C bridge linking the two monomers [39]. Therefore, it was concluded that **2** had the structure as shown, and the compound was given the trivial name aerimultin B.

Compound **3** was obtained as a brown amorphous solid. Its UV absorptions and IR absorption bands were similar to those of compound **2**, indicating a phenanthrene derivative. The HR-ESI-MS exhibited [M+Na]^+^ at *m*/*z* 533.1218 (calculated for C_30_H_22_O_8_Na, 533.1212), suggesting the molecular formula C_30_H_22_O_8._ However, the ^13^C NMR spectrum showed only 15 carbon signals, suggesting that **3** should be a dimeric phenanthrene with two identical units. Comparison of the ^1^H and ^13^C NMR of **3** with those of agrostonin (**8**) (Table 2) revealed their structural similarity, except for the presence of a hydroxyl group at C-6/C-6′ in **3**, instead of a methoxy group. Moreover, the two phenanthrene units were symmetrically linked to each other through a C−C bond between C-1 and C-1′ as supported by the HMBC correlations from C-1/C-1′ to H-3/H-3′, H-10/H-10′ and HO-2/HO-2′ [39]. On the basis of above spectral evidence, the structure of compound **3** was established as shown, and the trivial name aerimultin C was given to the compound.

Compound **4** was obtained as a yellow amorphous solid. The molecular formula was determined as C_21_H_26_O_7_ by HR-ESI-MS of [M+Na]^+^ at *m*/*z* 413.1584 (calculated for C_21_H_26_O_7_Na, 413.1576). The IR spectrum showed absorption bands for hydroxyl (3432 cm^−1^), aromatic ring (2937, 1608 cm^−1^), carbonyl ester (1723, 1208, 1111 cm^−1^) and methylene (1455 cm^−1^) groups. The UV spectrum exhibited maximum absorptions at 280 and 315 nm. The ^1^H NMR spectrum (Table 3) exhibited signals for a dihydroferulate structure [δ 2.59 (2H, t, *J* = 7.5 Hz, H_2_-8), 2.81 (2H, m, H_2_-7), 3.82 (3H, s, MeO-3), 6.68 (1H, dd, *J* = 8.1, 1.5 Hz, H-6), 6.73 (1H, d, *J* = 8.1 Hz, H-5), and 6.85 (1H, d, *J* = 1.5 Hz, H-2)] [40]. This was confirmed by the HMBC correlations of C-2 (δ 111.8), C-6 (δ 120.6) and C-9 (δ 172.2) with H_2_-7 (Table 3). The location of a MeO-3 group was supported by its NOESY correlation with H-2. The ^1^H NMR spectrum also showed signals for a dihydrosinapyl structure [δ 1.89 (2H, m, H_2_-8′), 2.57 (2H, t, *J* = 7.5 Hz, H_2_-7′), 3.80 (6H, s, MeO-3′, MeO-5′), 4.05 (2H, t, *J* = 7.5 Hz, H_2_-9′), and 6.49 (2H, s, H-2′, H-6′)] [41]. The HMBC correlations of C-2′/C-6′ (δ 105.8) and C-9′ (δ 63.2) with H_2_-7′ supported the presence of this unit.

The NOESY cross-peak between MeO-3′/MeO-5′ protons and H-2′/H-6′ confirmed the locations of the methoxy groups at C-3′/C-5′ (δ 147.7). The two phenylpropanoid units were connected by an ester bond at C-9 and C-9′, as determined by HMBC correlation of C-9 (δ 172.2) with H_2_-9′. Based on the above spectroscopic evidence, compound **4** was determined as dihydrosinapyl dihydroferulate. Prior to this study, the natural occurrence of **4** was unknown. However, the compound was earlier synthesized by acylation of the lignins from *Arabidopsis thaliana* [42].

### 2.2. Chemotaxonomic Significance

The presence of phenanthrene derivatives in *Aerides multiflora* is in line with the earlier findings in *A. crispum* and *A. rosea* [19,20]. In addition, the chemical profiles of these plants agreed with the conclusion from a molecular phylogenetic analysis that indicated their close relationships [43]. The family Orchidaceae is divided into 5 subfamilies, i.e., Epidendroideae, Orchidoideae, Vanilloideae, Cypripedioideae, and Apostasioideae [44]. So far, dimeric phenanthrenes have been reported from only two subfamilies, i.e., Epidendroideae (the genera *Stanhopea, Bletilla, Pholidota, Pleione, Otochilus, Arundina, Bulbophyllum, Dendrobium, Monomeria, Cremastra, Agrostophyllum, Liparis, Cyrtopodium*, *Eria*, *Eulophia*, *Cirrhopetalum*, *Calanthe, Lusia,* and *Prosthechea*) and Orchidoideae (the genera *Spiranthes* and *Gymnadenia*) [34,39,45,46,47,48,49,50,51,52,53,54,55,56,57,58,59,60,61,62,63]. No biphenanthrenes have been found outside these two subfamilies. Interestingly, a previous study revealed a monophyletic relationship between Epidendroideae and Orchidoideae [64]. Thus, from the currently available chemical data, the occurrence of biphenanthrenes could be taken as their chemotaxonomic marker. Nevertheless, additional chemical studies on the other three subfamilies are still needed to verify this postulation.

It should also be noted that the genus *Aerides* belongs to the same clade as *Rhynchostylis* [43]. Both genera have been called “the foxtail orchid” due to the erect or pendent inflorescences of closely packed flowers, and this has sometimes led to confusion. Up to the present, no reports on the secondary metabolites of the latter genus have appeared. Comparative chemical studies, in combination with detailed genetic analysis, may help shed light on the distinction between these two sister genera.

### 2.3. α-Glucosidase Inhibitory Activity

Yeast *α*-glucosidase enzyme was used in this study. In general, *α*-glucosidase enzymes can be obtained from several sources, for example, *Saccharomyces cerevisiae*, *Rattus norvegicus*, and GANC-human [65]. The enzyme derived from the yeast shows approximately 55% sequence homology with that obtained from mammalian sources [66], and therefore is widely employed in the investigations of natural compounds for *α*-glucosidase inhibitory potential [67,68].

All the isolated compounds (**1**–**10**) were initially tested for their *α*-glucosidase inhibitory activity at a concentration of 100 μg/mL. IC_50_ values were determined for compounds with more than 70% inhibition of the enzyme. As shown in Table 4, all compounds, except for dihydrosinapyl dihydroferulate (**4**), exhibited stronger activity (IC_50_ 5.2−266.7 μM) than the drug acarbose (IC_50_ value of 514.4 ± 9.2 μM). It should be mentioned that biphenanthrenes with *α*-glucosidase inhibitory activity were isolated from the family Orchidaceae for the first time in this study.

Overall, the dimeric phenanthrenes (**1**, **2**, **3**, and **8**) demonstrated higher activity than the monomers (**5**, **7**, and **10**), as indicated by their IC_50_ values (Table 4). Aerimultin C (**3**) was the most potent *α*-glucosidase inhibitor, with an IC_50_ value of 5.2 ± 0.7 μM. Replacing the phenolic groups at C-6 and C-6′ of this compound with methoxy groups reduced the activity by about seven-fold, as can be seen from the increased IC_50_ value (37.2 ± 4.5 μM) for agrostonin (**8**). The importance of free OH groups is also supported by the potent activity (IC_50_ 2.08 ± 0.19 μM) earlier observed for a biphenanthrene (from *Dioscorea bulbifera*, Dioscoreaceae), the structure of which contains four free phenolic groups [69]. A molecular docking study on flavones with *α*-glucosidase inhibitory activity has also revealed that replacement of the hydroxyl groups with methoxy groups could lead to loss of activity [70].

Parallel observations were also obtained for the 3-phenylpropyl 3-propionate derivatives (**4** and **9**). Dihydroconiferyl dihydro-*p*-coumarate (**9**) showed appreciable activity (IC_50_ 266.7 ± 8.6 μM). However, introducing methoxy groups to C-3 and C-5′ of **9** caused a total loss of activity, as seen in dihydrosinapyl dihydroferulate (**4**). A similar phenomenon, in which the presence of aromatic methoxy groups diminished *α*-glucosidase inhibitory activity, was earlier reported for *p*-coumarate esters of long-chain alcohols [71].

**Figure 3:**
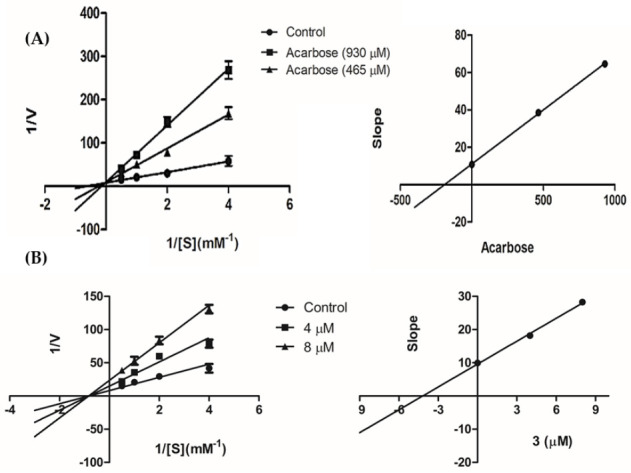
Lineweaver–Burk plots of (**A**) acarbose and (**B**) compound **3**. The secondary plot of each compound is on the right.

A kinetics study was then performed on compound **3** to analyze the mode of enzyme inhibition using various substrate concentrations (0.25–2.0 mM). From the Lineweaver–Burk plot in Figure 3A, the drug acarbose showed the intersection of the lines on the *y*-axis, indicating competitive type of inhibition. The K*_i_* value of acarbose (190.57 μM) was obtained from the secondary plot by replotting the slopes of the lines against inhibitor concentrations. For compound **3**, the increase in concentration (4 and 8 μM) decreased the V*_max_* from 0.10 to 0.035 but did not affect the K*_m_* value (Figure 3B). The results suggested non-competitive inhibition of the enzyme by **3**. The K*_i_* value of compound **3** (4.18 μM) was obtained from the secondary plot, as shown in Table 5**.**

Generally, non-competitive inhibitors have some advantages over competitive inhibitors [72]. Non-competitive inhibitors bind to the allosteric site of the enzyme, and thus do not depend upon the substrate concentration. Moreover, they require lower concentrations than competitive inhibitors to produce the same effect [73]. Compound **3**, as a potent non-competitive inhibitor of *α*-glucosidase, provides a lead structure for the further design and development of AGI drugs.

## 3. Materials and Methods

### 3.1. Experimental

Optical rotations were determined with a PerkinElmer Polarimeter 341 (Boston, MA, USA). UV spectra were recorded on a Milton Roy Spectronic 3000 Array spectrophotometer (Rochester, Monroe, NY, USA). IR spectra were obtained with a PerkinElmer FT-IR 1760X spectrophotometer (Boston, MA, USA). Mass spectra were measured using a Bruker MicroTOF mass spectrometer (ESI-MS) (Billerica, MA, USA). NMR spectra were recorded on a Bruker Avance DPX-300FT NMR spectrometer or a Bruker Avance III HD 500 NMR spectrometer (Billerica, MA, USA). Yeast *α*-glucosidase enzyme and *p*-nitrophenol-*α*-d-glucopyranoside were obtained from Sigma Chemical, Inc. (St. Louis, MO, USA), and acarbose was purchased from Fluka Chemical (Buchs, Switzerland). Microtiter plate readings were carried out with a CLARIOstar apparatus (BMGLABTECH, Ortenberg, Germany).

### 3.2. Plant Material

The plant materials, whole plants of *Aerides multiflora*, were purchased from Chatuchak market in May 2019. Plant identification was performed by Mr. Yanyong Punpreuk, Department of Agriculture, Bangkok, Thailand. A voucher specimen BS-AM-052562 has been deposited at the Department of Pharmacognosy and Pharmaceutical Botany, Faculty of Pharmaceutical Sciences, Chulalongkorn University.

### 3.3. Extraction and Isolation

The dried powder from the whole plants of *Aerides multiflora* (6.1 kg) was macerated with MeOH (4 × 18 L). The MeOH extract, at a concentration of 100 µg/mL, showed 82.4 ± 9.5% inhibition of *α*-glucosidase. This MeOH extract (550 g) was then suspended in water and partitioned with EtOAc and butanol to give an EtOAc extract (201.1 g), a butanol extract (80.8 g), and an aqueous extract (150 g), respectively. The EtOAc extract exhibited 92.9 ± 3.2 inhibition at 100 µg/mL, whereas the others were devoid of activity (<50% inhibition). Therefore, the EtOAc extract was subjected to further investigation.

The EtOAc extract was first fractionated by vacuum liquid chromatography (silica gel, EtOAc–CH_2_Cl_2_, gradient) to give five fractions (A–E). Fraction B (11.4 g) was further fractionated on a silica gel column (EtOAc–hexane, gradient) to give 3 fractions (BA–BC). Fraction BA (1 g) was separated on Sephadex LH-20 (methanol) to yield fractions BAA, BAB, and BAC. Fraction BAA (200 mg) was further separated by column chromatography (CC, silica gel, EtOAc–CH_2_Cl_2_, gradient) to give 6-methoxycoelonin (**5**) (65.4 mg). Fraction BAB (300 mg) was subjected to CC (silica gel, EtOAc–CH_2_Cl_2_, gradient) to give fractions BAB1 and BAB2. Fraction BAB1 (160.2 mg) was separated by CC (silica gel, acetone-hexane, 3:7) to yield **1** (2.3 mg). Gigantol (**6**) (14.5 mg) was obtained from fraction BAB2 (100 mg) after purification on Sephadex LH-20 (acetone). Fraction BB (1 g) was separated on Sephadex LH-20 (acetone) to yield BBA and BBB fractions. Fraction BBA (195.8 mg) was subjected to CC (silica gel, EtOAc-CH_2_Cl_2_, gradient) to yield BBA1 and BBA2 fractions. Fraction BBA1 (132.2 mg) was subjected to CC (silica gel, acetone–hexane, 3:7) to produce imbricatin (**7**) (39 mg) and agrostonin (**8**) (7 mg). Fraction C (10.5 g) was fractionated on a silica gel column (EtOAc-CH_2_Cl_2_, gradient) to give 3 fractions (CA–CC). Fraction CB (500 mg) was further separated on Sephadex LH-20 (acetone) to yield CBA and CBB fractions. Fraction CBA (236.9 mg) was further separated by CC (silica gel, EtOAc–hexane, gradient) to give dihydroconiferyl dihydro-*p*-coumarate (**9**) (74.1 mg) and 5-methoxy-9,10-dihydrophenanthrene-2,3,7-triol (**10**) (9.2 mg). Fraction CC (100 mg) was separated on Sephadex LH-20 (acetone) to yield fractions CCA, CCB, and CCC. Fraction CCB (10 mg) was subjected to CC (silica gel, EtOAc–CH_2_Cl_2_, 0.2: 9.8) to yield **2** (3.9 mg). Fraction D (72 g) was chromatographed on a silica gel column (EtOAc-CH_2_Cl_2_, gradient) to give 3 fractions (DA–DC). Fraction DA (1 g) was separated on Sephadex LH-20 (methanol) to yield DAA and DAB fractions. Fraction DAA (300 mg) was re-separated on Sephadex LH20 (acetone) to yield DAA1 and DAA2 fractions. Fraction DAA1 (100 mg) was subjected to CC (silica gel, EtOAc–hexane, 3:7) to give **4** (4.2 mg). Fraction E (84.8 g) was subjected to Diaion HP-20 (water–methanol, gradient) to yield five fractions (EA–EE). Fraction EC (1.7 g) was separated on Sephadex LH-20 (methanol) to yield ECA, ECB and ECC fractions. Fraction ECC (40 mg) was subjected to CC (silica gel, methanol–CH_2_Cl_2_, 0.5:9.5) to produce **3** (8.8 mg).

Aerimultin A (**1**): whitish-brown amorphous powder; UV (MeOH) λ_max_ (log ε) 265 (4.31), 305 (4.2), 315 (4.19); IR: ν_max_ 3350, 2923, 2850, 1696, 1605, 1462, 1442, 1221, 1201 cm^−1^; HR-ESI-MS: [M+Na]^+^ at *m*/*z* 565.1841 (calculated for C_32_H_30_O_8_Na, 565.1838); ^1^H and ^13^C NMR data, see Table 1.

Aerimultin B (**2**): brown amorphous solid; [*α*] _D_^20^ −108 (*c* 0.005, MeOH); UV (MeOH) λ_max_ (log ε) 265 (4.67), 315 (4.09), 370 (3.99); IR: ν_max_ 3368, 2919, 2850, 1736, 1587, 1463, 1259 cm^−1^; HR-ESI-MS: [M+Na]^+^ at *m*/*z* 559.1376 (calculated for C_32_H_24_O_8_Na, 559.1368); ^1^H and ^13^C NMR data, see Table 2.

Aerimultin C (**3**): brown amorphous solid; [*α*] _D_^20^ +67.5 (*c* 0.008, MeOH); UV (MeOH) λ_max_ (log ε) 265 (4.1), 315 (3.42), 355 (3.47), 370 (3.48); IR: ν_max_ 3360, 2921, 2851, 1712, 1588, 1461, 1371 cm^−1^; HR-ESI-MS: [M+Na]^+^ at *m*/*z* 533.1218 (calculated for C_30_H_22_O_8_Na, 533.1212); ^1^H and ^13^C NMR data, see Table 2.

Dihydrosinapyl dihydroferulate (**4**): yellow amorphous solid; UV (MeOH) λ_max_ (log ε) 280 (3.76), 315 (3.12); IR: ν_max_ 3432, 2937, 2841, 1723, 1608, 1514, 1455, 1427, 1208, 1111 cm^−1^; HR-ESI-MS: [M+Na]^+^ at *m*/*z* 413.1584 (calculated for C_21_H_26_O_7_Na, 413.1576); ^1^H and ^13^C NMR data, see Table 3.

### 3.4. α-Glucosidase Inhibition Assay

The assays were performed following previous protocols [74]. The liberation of *p*-nitrophenol from the substrate *p*-nitrophenol-*α*-d-glucopyranoside (PNPG) was observed to determine the inhibition of the *α*-glucosidase enzyme. Each sample was initially dissolved in 50% DMSO. Then, 0.1 U/mL of *α*-glucosidase (40 μL) in phosphate buffer (pH 6.8) was added to each well of a 96-well plate which contained the sample solution (10 μL). The plate was pre-incubated at 37 °C for 10 min. Then, 2 mM *p*-nitrophenol-*α*-d-glucopyranoside (50 μL) was added, and the mixture was incubated again at 37 °C for 20 min. Finally, 1 M Na_2_CO_3_ solution (100 μL) was added to terminate the reaction. The absorbance of the mixture was measured at 405 nm using a microplate reader. Two-fold serial dilution was performed for IC_50_ determination. The drug acarbose was used as the positive control.

The mode of enzyme inhibition of the test compound was determined using the double reciprocal Lineweaver–Burk plot (1/V vs. 1/[S]). The experiment was performed by varying the PNPG concentrations (0.25, 0.5, 1.0, and 2.0 mM) in the absence or presence of compound **3** (4 μM and 8 μM) or acarbose (930 μM and 465 μM). The secondary plot was constructed by replotting the slopes of the lines against inhibitor concentrations, and the K*_i_* was calculated from the line equation of the plot.

## 4. Conclusions

In this communication, ten compounds were isolated from *Aerides multiflora*, including three new compounds, namely, aerimultins A–C (**1**–**3**), the new natural product dihydrosinapyl dihydroferulate (**4**), and six known compounds (**5**–**10**). The structures of the new compounds were established by spectroscopic methods. The findings in this study suggested that biphenanthrenes might be taken as a chemotaxonomic marker for the subfamilies Epidendroideae and Orchidoideae within the family Orchidaceae. For the first time, the dimeric phenanthrenes obtained from this plant family were investigated for an *α*-glucosidase inhibitory activity. Among the isolates, the biphenathrene aerimultin (**3**) emerged as the most potent inhibitor, showing much higher potency than the drug acarbose. An enzyme kinetic study on this compound revealed a non-competitive type of inhibition and suggested that it could be a candidate structure for *α*-glucosidase inhibitor drug development.

## Figures and Tables

**Table 1:** ^1^H (500 MHz) and ^13^C-NMR (125 MHz) spectral data of **1** and **5** in acetone-*d*_6_.

Position	1 ^a^			5 ^b^	
	δ_H_ (Multiplicity, *J* in Hz)	δ_C_	HMBC (Correlation with ^1^H)	δ_H_ (Multiplicity, *J* in Hz)	δ_C_
1	6.35 (d, *J* = 2.5 Hz)	108.3	3, 10, HO-2	6.39 (br s)	107.4
2	-	157.8	1 *, 3 *, HO-2 *	-	156.5
3	6.46 (d, *J* = 2.5 Hz)	99.1	1, HO-2	6.65 (br s)	98.3
4	-	158.8	3 *, MeO-4	-	157.7
4a	-	115.9	1, 3, 5, 10	-	115.5
4b	-	127.8	5 *, 8, 9	-	124.7
5	7.98 (s)	114.3	-	7.89 (s)	112.2
6	-	147.4	8, MeO-6	-	145.1
7	-	146.6	5	-	144.3
8	6.33 (s)	113.6	9	6.69 (s)	114.0
8a	-	131.1	5, 10	-	130.7
9	2.45 (m)	29.0	8	2.61 (m)	28.9
10	2.56 (m)	31.4	1	2.61 (m)	30.7
10a	-	141.6	9, 10 *	-	140.5
1′	-	133.7	3′, 10′, HO-2′		
2′	-	149.8	3′ *, HO-2′ *		
3′	6.65 (s)	100.2	HO-2′		
4′	-	155.3	3′ *, MeO-4′		
4a′	-	117.1	3′, 5′, 10′		
4b′	-	125.2	5′ *, 8′, 9′		
5′	7.93 (s)	113.4	-		
6′	-	146.1	8′, MeO-6′, HO-7′		
7′	-	145.6	5′, HO-7′ *		
8′	6.66 (s)	114.9	9′, HO-7′		
8a′	-	131.4	5′, 10′		
9′	2.52 (br s)	29.9	8′		
10′	2.52 (br s)	24.1	-		
10a′	-	133.8	9′		
MeO-4	3.89 (s)	56.1	-	3.86 (s)	55.5
MeO-6	3.92 (s)	56.5	-	3.83 (s)	54.9
MeO-4′	3.91 (s)	56.4	-		
MeO-6′	3.84 (s)	55.8	-		
HO-2	8.35 (s)	-	-		
HO-2′	8.25 (s)	-	-		
HO-7′	7.44 (s)	-	-		

^a 1^H (500 MHz) and ^13^C-NMR (125 MHz); ^b 1^H (300 MHz) and ^13^C-NMR (75 MHz); * two-bond coupling.

**Table 2:** ^1^H and ^13^C-NMR spectral data of **2, 3** and **8** in acetone-*d*_6_.

Position	2 ^a^			3 ^b^			8 ^b^	
	δ_H_ (Multiplicity, *J* in Hz)	δ_C_	HMBC (Correlation with ^1^H)	δ_H_ (Multiplicity, *J* in Hz)	δ_C_	HMBC (Correlation with ^1^H)	δ_H_ (Multiplicity, *J* in Hz)	δ_C_
1	-	109.3	3, 10	-	108.8	3, 10, HO-2	-	108.9
2	-	155.0	3 *	-	154.1	3 *, HO-2 *	-	154.2
3	6.99 (s)	100.0	-	6.95 (s)	98.8	HO-2	7.00 (s)	99.2
4	-	160.2	3 *, MeO-4	-	159.4	3 *, MeO-4	-	159.3
4a	-	116.2	3, 5, 10	-	115.1	3, 5, 10	-	115.5
4b	-	125.8	8, 9	-	125.3	8, 9	-	124.9
5	9.24 (s)	109.8	-	9.19 (s)	112.7	-	9.26 (s)	111.3
6	-	148.5	5 *, 8, MeO-6	-	145.3	8	-	147.6
7	-	146.0	5, 8 *	-	144.1	5	-	145.2
8	7.19 (s)	112.2	9	7.19 (s)	111.5	9	7.20 (s)	112.3
8a	-	128.0	5, 10	-	126.7	5, 10	-	127.2
9	7.36 (d, *J* = 9.5 Hz)	126.5	8	7.31 (d, *J* = 9.0 Hz)	127.2	8	7.37 (d, *J* = 9.0 Hz)	127.1
10	6.98 (d, *J* = 9.5 Hz)	123.3	-	6.87 (d, *J* = 9.0 Hz)	121.8	-	6.95 (d, *J* = 9.0 Hz)	122.5
10a	-	135.4	9	-	134.6	9	-	134.6
1′	-	110.2	3′, 10′	-	108.8	3′, 10′, HO-2′	-	108.9
2′	-	156.3	3′ *	-	154.1	3′ *, HO-2′ *	-	154.2
3′	6.81 (s)	103.1	-	6.95 (s)	98.8	HO-2′	7.00 (s)	99.2
4′	-	153.7	3′ *, 11′	-	159.4	3′ *, MeO-4′	-	159.3
4a′	-	113.0	3′, 10′	-	115.1	3′, 5′, 10′	-	115.5
4b′	-	119.1	8′, 9′, 11′	-	125.3	8′, 9′	-	124.9
5′	-	120.6	11′ *	9.19 (s)	112.7	-	9.26 (s)	111.3
6′	-	144.2	8′, 11′, MeO-6′	-	145.3	8′	-	147.6
7′	-	150.3	8′ *	-	144.1	5′	-	145.2
8′	7.21 (s)	111.6	9′	7.19 (s)	111.5	9′	7.20 (s)	112.3
8a′	-	126.2	10′	-	126.7	5′, 10′	-	127.2
9′	7.37 (d, *J* = 9.0 Hz)	127.9	8′	7.31 (d, *J* = 9.0 Hz)	127.2	8′	7.37 (d, *J* = 9.0 Hz)	127.1
10′	6.92 (d, *J* = 9.0 Hz)	124.6	-	6.87 (d, *J* = 9.0 Hz)	121.8	-	6.95 (d, *J* = 9.0 Hz)	122.5
10a′	-	132.2	9′	-	134.6	9′	-	134.6
11′	5.79 (d, *J* = 1.5 Hz)	64.8	-	-	-	-	-	-
MeO-4	4.22 (s)	56.1	-	4.18 (s)	55.0		4.24 (s)	55.3
MeO-6	4.06 (s)	56.0	-	-	-		4.08 (s)	55.2
MeO-4′	-	-	-	4.18 (s)	55.0		4.24 (s)	55.3
MeO-6′	3.95 (s)	61.3	-	-	-		4.08 (s)	55.2
HO-2	-	-	-	7.54 (s)	-		7.61 (s)	-
HO-2′	-	-	-	7.54 (s)	-		7.61 (s)	-

^a 1^H (500 MHz) and ^13^C-NMR (125 MHz); ^b 1^H (300 MHz) and ^13^C-NMR (75 MHz); * two-bond coupling.

**Table 3:** ^1^H (300 MHz) and ^13^C-NMR (75 MHz) spectral data of **4** in acetone-*d*_6._

Position	δ_H_ (Multiplicity, *J* in Hz)	δ_C_	HMBC (Correlation with ^1^H)
1	-	132.1	5, 7 *, 8
2	6.85 (d, *J* = 1.5 Hz)	111.8	6, 7
3	-	147.3	5, MeO-3, HO-4
4	-	144.9	2, 6, HO-4 *
5	6.73 (d, *J* = 8.1 Hz)	114.8	HO-4
6	6.68 (dd, *J* = 8.1, 1.5 Hz)	120.6	2, 7
7	2.81 (m)	30.4	2, 6, 8 *
8	2.59 (t, *J* = 7.5 Hz)	35.8	7 *
9	-	172.2	7, 8 *, 9′
1′	-	131.7	8′
2′	6.49 (s)	105.8	6′, 7′
3′	-	147.7	2′ *, HO-4′, MeO-3′
4′	-	134.2	2′, 6′, HO-4′ *
5′	-	147.7	6′ *, HO-4′, MeO-5′
6′	6.49 (s)	105.8	2′, 7′
7′	2.57 (t, *J* = 7.5 Hz)	31.8	2′, 6′, 8′ *, 9′
8′	1.89 (m)	30.4	7′ *, 9′ *
9′	4.05 (t, *J* = 7.5 Hz)	63.2	7′, 8′ *
MeO-3	3.82 (s)	55.3	-
MeO-3′	3.80 (s)	55.7	-
MeO-5′	3.80 (s)	55.7	-
HO-4	7.35 (s)	-	-
HO-4′	6.94 (s)	-	-

* Two-bond coupling.

**Table 4:** *α*-Glucosidase inhibitory activity of compounds **1**–**10**.

Compounds	IC_50_ (μg/mL)	IC_50_ (μM)
Aerimultin A (**1**)	16.8 ± 1.0	30.9 ± 1.9
Aerimultin B (**2**)	41.8 ± 1.3	77 ± 2.5
Aerimultin C (**3**)	2.7 ± 0.4	5.2 ± 0.7
Dihydrosinapyl dihydroferulate (**4**)	NA	NA
6-Methoxy coelonin (**5**)	61.2 ± 2.2	224.8 ± 7.8
Gigantol (**6**)	52.5 ± 1.9	191.3 ± 6.8
Imbricatin (**7**)	44.9 ± 2.1	165.9 ± 7.7
Agrostonin (**8**)	20.1 ± 2.5	37.2 ± 4.5
Dihydroconiferyl dihydro-*p*-coumarate (**9**)	88.1 ± 2.9	266.7 ± 8.6
5-Methoxy-9,10-dihydrophenanthrene-2,3,7-triol (**10**)	29.7 ± 2.3	115.2 ± 9.1
Acarbose	332.1 ± 5.9	514.4 ± 9.2

NA = no inhibitory activity.

**Table 5:** Kinetic parameters of *α*-glucosidase inhibition in the presence of **3**.

Inhibitors	Dose (μM)	V*_max_* ∆OD/min	K*_m_* (mM)	K*_i_* (μM)
None	-	0.10	1.22	
3	8	0.035	1.20	4.18
	4	0.055	1.22	
Acarbose	930	0.11	6.47	190.57
	465	0.10	4.17	

V*_max_*, maximum rate of velocity; K*_m_*, Michaelis constant; K*_i_*, inhibitor constant.

## Data Availability

All data presented in this study are available in the article.
